# Microbial α-L-Rhamnosidases: Regioselective Biocatalysts for Flavonoid Biotransformation and Nutraceutical Applications

**DOI:** 10.3390/cimb48060625

**Published:** 2026-06-16

**Authors:** Massimo Iorizzo

**Affiliations:** Department of Agricultural, Environmental and Food Sciences, University of Molise, Via De Sanctis, 86100 Campobasso, Italy; iorizzo@unimol.it

**Keywords:** α-L-rhamnosidase, GH78, flavonoid biotransformation, selective deglycosylation, nutraceuticals, microbiota, food biotechnology

## Abstract

Microbial α-L-rhamnosidases are increasingly recognised as selective biocatalysts in food biotechnology, nutraceutical production, and health-related applications. These glycoside hydrolases catalyse the hydrolysis of terminal alpha-L-rhamnose residues from flavonoids, terpenoids, saponins, and other glycosylated natural products, thereby modulating sensory properties, solubility, intestinal absorption, and biological activity. While their traditional uses include debittering citrus juice and enhancing wine aroma, recent evidence demonstrates their wider value in selective flavonoid biotransformation, production of rare mono-glycosylated derivatives, probiotic fermentations, and microbiome-associated metabolism. This review summarises microbial sources, catalytic mechanisms, CAZy classification, substrate specificity, structure–function relationships, analytical methods, industrial process engineering, and emerging applications in functional foods and targeted nutraceutical applications. Particular attention is given to the distinction between alpha-(1→2)- and alpha-(1→6)-linked substrates, the production of isoquercitrin and prunin, recombinant enzyme platforms, immobilised biocatalysts, and potential future opportunities arising from metagenomics, synthetic biology, and AI-assisted protein engineering.

## 1. Introduction

Microbial α-L-rhamnosidases (EC 3.2.1.40) are glycoside hydrolases that catalyse the cleavage of terminal alpha-L-rhamnose residues from natural glycosides, oligosaccharides, and glycoconjugates [1,2,3]. They are found in bacteria, yeasts, filamentous fungi, and extremophilic microorganisms, where they are involved in the degradation of plant-derived carbohydrates, flavonoid glycosides, pectic fragments, and secondary metabolites [1,2,4,5]. Representative microbial producers of α-L-rhamnosidases and their main biochemical characteristics are summarised in Table 1. The increasing interest in these enzymes stems from their ability to carry out selective transformations under mild conditions, making them suitable for food, nutraceutical, cosmetic, and pharmaceutical applications [1,2,6].

Rhamnose-containing compounds are abundant in fruits, vegetables, medicinal plants, fermented foods, and agro-industrial residues. Relevant substrates include flavonoid rutinosides and neohesperidosides, terpenyl glycosides, saponins, rhamnolipids, and complex plant polysaccharide fragments [1,2,6]. Notably, rutin, hesperidin, naringin, neohesperidin, narirutin, and eriocitrin have attracted particular attention because their glycosylation patterns strongly influence bitterness, water solubility, stability, absorption, and biological activity [7,8,9].

The selective removal of rhamnose can produce mono-glycosylated derivatives such as isoquercitrin and prunin, which often exhibit improved bioavailability or altered bioactivity compared with their parent diglycosides [10,11]. Historically, microbial α-L-rhamnosidases have been associated mainly with citrus juice debittering and wine aroma enhancement [12,13]. In citrus processing, naringinase preparations hydrolyse naringin, a major bitter flavanone glycoside, while in winemaking, rhamnosidases work with other glycosidases to release volatile terpenes from odourless glycosylated precursors [4,11]. More recently, these enzymes have been recognised as highly selective biocatalysts for controlled flavonoid engineering, selective nutraceutical production, and microbiome-oriented functional food design [14].

Food-grade and probiotic rhamnosidases, particularly those produced by *Lactiplantibacillus plantarum* and related lactic acid bacteria (LAB), represent an important emerging area [10,11,15]. Humans lack endogenous α-L-rhamnosidases capable of efficiently hydrolysing many dietary flavonoid rhamnosides; therefore, microbial metabolism is essential for the release of absorbable glucosides and aglycones in the gastrointestinal tract [16]. These findings highlight microbial rhamnosidases as key contributors to diet–microbiome interactions and interindividual variability in polyphenol metabolism [14,16].

This review examines microbial α-L-rhamnosidases as selective biocatalysts for food, nutraceutical, and health applications. It highlights catalytic mechanisms, microbial diversity, genetic regulation, substrate specificity, analytical methods, applications in food and beverages, probiotic systems, recombinant production, industrial process engineering, and future perspectives in precision biocatalysis. The literature included in this review was collected from PubMed, Scopus, Web of Science, and Google Scholar databases using combinations of the keywords “α-L-rhamnosidase”, “GH78”, “flavonoid biotransformation”, “naringinase”, “probiotics”, “microbiota”, and “nutraceuticals”.

**Table 1:** Representative Microbial Sources and Biochemical Properties of α-L-Rhamnosidases.

Microorganism	Type	GH Family	Optimum pH	Optimum Temp (°C)	Preferred Substrate	Main Application	References
*Aspergillus niger*	Filamentous fungus	GH78	4–5	50–60	Naringin	Citrus debittering	[1,7,17]
*Pichia angusta*	Yeast	GH78	6	40	Rutin	Flavonoid hydrolysis	[2,4]
*Clavispora lusitaniae*	Yeast	GH78	4	50	Hesperidin	Acidic beverages	[2,18]
*Lactiplantibacillus plantarum*	LAB	GH78	5–7	50–60	Rutin/Hesperidin	Probiotic biotransformation	[5,10,11]
*Dictyoglomus thermophilum*	Thermophile	GH78	6–7	95	Naringin	Thermostable biocatalysis	[6,19]
*Bacillus* sp.	Bacterium	GH78	6–8	45–60	Flavonoids	Industrial hydrolysis	[2,20]

Several reviews have previously addressed α-L-rhamnosidases from specific perspectives, including enzyme production, biochemical characterization, industrial applications, or microbial sources. However, recent advances in structural biology, flavonoid regioselective biotransformation, microbiota-associated metabolism, recombinant engineering, and precision biocatalysis have significantly expanded the scientific and technological relevance of these enzymes. To date, no comprehensive review has integrated these emerging developments within a unified framework focused on microbial α-L-rhamnosidases as selective biocatalysts for flavonoid transformation and nutraceutical production. The present review aims to fill this gap by critically examining microbial diversity, catalytic mechanisms, substrate selectivity, food-grade applications, nutraceutical relevance, industrial biocatalysis, and future engineering strategies while highlighting current limitations and future research priorities.

### Literature Search Strategy

The literature included in this review was systematically collected from PubMed, Scopus, Web of Science, and Google Scholar databases up to March 2026. Searches were performed using combinations of the keywords “α-L-rhamnosidase”, “GH78”, “GH106”, “flavonoid biotransformation”, “naringinase”, “probiotic”, “gut microbiota”, “nutraceutical”, “deglycosylation”, and “flavonoid glycosides”.

Inclusion criteria comprised peer-reviewed research articles, reviews, structural biology studies, enzymatic characterization reports, recombinant expression studies, and industrial application papers directly related to microbial α-L-rhamnosidases. Particular attention was given to publications describing substrate specificity, catalytic mechanisms, structure–function relationships, food applications, probiotic metabolism, and emerging biotechnological developments.

Conference abstracts, duplicate publications, non-peer-reviewed documents, and studies lacking sufficient experimental characterization were excluded whenever possible. Priority was given to publications from 2000–2026, although seminal earlier studies were included when relevant for understanding catalytic mechanisms, enzyme classification, and historical development of the field.

The selected literature was critically evaluated to identify current advances, unresolved challenges, and future perspectives for the application of microbial α-L-rhamnosidases in food biotechnology and nutraceutical production.

## 2. Biochemistry and Catalytic Mechanisms of α-L-Rhamnosidases

α-L-rhamnosidases are mainly classified within GH78 and GH106 families, with GH78 representing the most extensively studied group for biotechnological applications [1,21]. Although both GH78 and GH106 enzymes catalyse the hydrolysis of terminal α-L-rhamnosyl residues through an inverting mechanism, important differences exist in their structural organization, substrate preferences, and biotechnological relevance. GH78 enzymes represent the largest and best-characterized group, with numerous structural studies and demonstrated applications in flavonoid biotransformation, food processing, and nutraceutical production. In contrast, GH106 enzymes remain comparatively less studied, with fewer available structures and more limited information regarding their substrate specificity and industrial potential. Consequently, most current biotechnological developments involving α-L-rhamnosidases are based on GH78 enzymes.

The principal biochemical, structural, and biotechnological differences between GH78 and GH106 α-L-rhamnosidases are summarized in Table 2.

**Figure 1:**
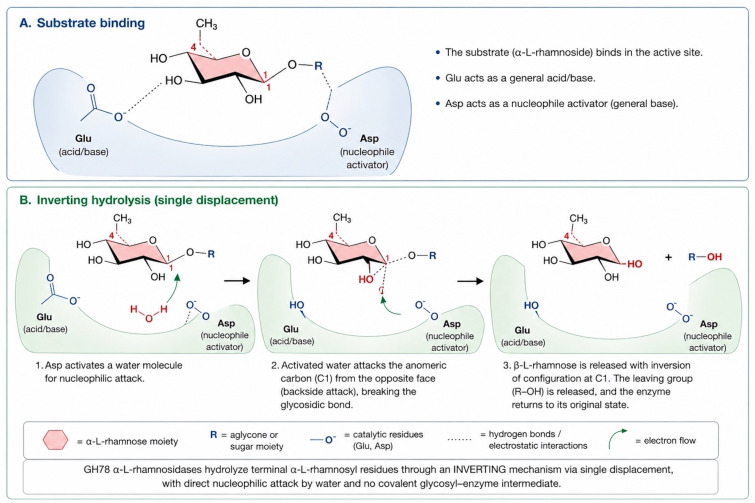
Schematic representation of the inverting catalytic mechanism of microbial GH78 α-L-rhamnosidases involved in the hydrolysis of terminal α-L-rhamnose residues from flavonoid glycosides. Original figure created by the author.

GH78 enzymes hydrolyse terminal alpha-L-rhamnosyl residues through an inverting mechanism, releasing beta-L-rhamnose without formation of a covalent glycosyl–enzyme intermediate [3]. This catalytic mechanism typically involves two acidic residues acting as the catalytic acid and base, with additional residues stabilising the rhamnose moiety and determining substrate orientation [22,23]. The main structural and catalytic features of α-L-rhamnosidases are shown schematically in Figure 1.

Structural studies have shown that GH78 α-L-rhamnosidases often exhibit multidomain architectures combining catalytic and auxiliary carbohydrate-binding or dimerisation domains [22,23]. Representative biochemical and structural features of microbial α-L-rhamnosidases are summarised in Table 3. The first GH78 structure was determined for *Bacillus* sp. GL1 RhaB, followed by structures from *Bacteroides thetaiotaomicron*, *Streptomyces avermitilis*, *Klebsiella oxytoca*, and *D. thermophilum* [6,20,22,23]. These structures revealed deep substrate-binding clefts capable of accommodating bulky flavonoid glycosides and provided a molecular basis for linkage selectivity and aglycone recognition [11,14]. Substrate selectivity is influenced by glycosidic linkage, aglycone structure, active-site topology, and hydrogen-bond networks [6,22,23]. Some enzymes prefer alpha-(1→2)-linked neohesperidosides such as naringin and neohesperidin, while others preferentially hydrolyse alpha-(1→6)-linked rutinosides such as rutin and hesperidin [5,6]. For example, DtRha from *D. thermophilum* preferentially derhamnosylates alpha-(1→2)-linked flavonoids, whereas L. plantarum enzymes often show higher activity towards alpha-(1→6)-linked substrates [6,10]. Biochemical properties differ significantly among microbial sources. LAB enzymes typically have optima at mildly acidic to neutral pH and moderate temperatures, while thermophilic enzymes remain active at very high temperatures and in organic solvent mixtures [6,10]. Acid stability, thermostability, solvent tolerance, and lack of undesired beta-glucosidase activity are especially important for citrus juices, wine, and selective flavonoid biotransformation [12,24].

## 3. Microbial Diversity of α-L-Rhamnosidases

Microbial α-L-rhamnosidases are present in filamentous fungi, yeasts, LAB, soil bacteria, actinomycetes, marine microorganisms, and thermophiles [1,3].

**Figure 2:**
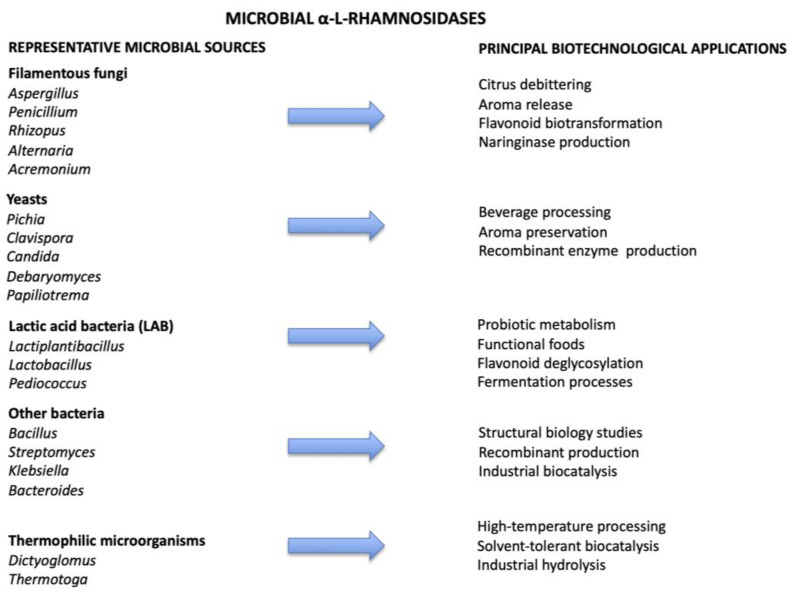
Major microbial sources of α-L-rhamnosidases and their principal industrial and nutraceutical applications. Original figure created by the author.

The major microbial groups producing α-L-rhamnosidases and their principal biotechnological applications are summarized in Figure 2. This overview highlights the diversity of microbial sources currently exploited for food processing, nutraceutical production, probiotic biotechnology, and industrial biocatalysis. This diversity reflects adaptation to plant-rich environments and the widespread presence of rhamnose-containing substrates in plant cell walls, flavonoids, and secondary metabolites [1,18].

Filamentous fungi remain important industrial sources of α-L-rhamnosidase and naringinase preparations. Species of *Alternaria*, *Acremonium*, *Aspergillus*, *Penicillium*, and *Rhizopus* have been studied for enzyme production, citrus debittering, aroma release, and flavonoid modification [1,17,25,26,27,28]. *Aspergillus niger* and *Penicillium decumbens* are particularly significant, as commercial naringinase and hesperidinase preparations are typically derived from fungal sources and contain both α-L-rhamnosidase and beta-D-glucosidase activities [7]. Yeast-derived α-L-rhamnosidases have received less attention but offer important advantages, including short fermentation cycles, food compatibility, and potential aroma preservation. *Pichia*, *Clavispora*, *Candida*, *Debaryomyces*, *Hansenula*, *Cryptococcus*, and *Papiliotrema* have been reported as producers of rhamnosidase activity [18,29,30,31]. Several yeast enzymes are promising for use in acidic beverages and fruit matrices, although structural information remains limited compared with that available for bacterial GH78 enzymes [2,4]. Bacterial α-L-rhamnosidases are highly diverse and include enzymes from *Lactiplantibacillus*, *Lactobacillus*, *Pediococcus*, *Bifidobacterium*, *Bacillus*, *Streptomyces*, *Klebsiella*, *Escherichia*, *Sphingomonas*, *Dictyoglomus* and some thermophilic bacteria [5,14,23,32,33,34]. LAB are especially important for food-grade applications and probiotic flavonoid metabolism [5,10,15,35].

Human gut bacteria also encode GH78 enzymes with diverse substrate selectivity for dietary flavonoid diglycosides, supporting their role in polyphenol metabolism [14,16]. Thermophilic and extremophilic microorganisms provide enzymes with high operational robustness. DtRha from *Dictyoglomus thermophilum* is a representative thermophilic GH78 enzyme with high temperature tolerance, solvent compatibility, a resolved structure, and strong selectivity for alpha-(1→2)-linked flavonoids [6,19]. These properties make extremophilic enzymes attractive for processing poorly soluble flavonoids, use in non-aqueous systems, and high-temperature industrial processes [2,6]. Filamentous fungi are the most extensively studied source of industrial α-L-rhamnosidases.

Table 1 offers a comparative overview of microbial producers of α-L-rhamnosidases, including filamentous fungi, yeasts, LAB, and thermophilic microorganisms. It also summarises glycoside hydrolase family classification, biochemical properties, preferred substrates, and principal industrial or nutraceutical applications.

### Emerging Engineering Strategies

Recent studies have highlighted the increasing interest in microbial α-L-rhamnosidases displaying enhanced thermostability, acid tolerance, solvent resistance, and regioselective flavonoid biotransformation capabilities. In particular, novel GH78 enzymes from thermophilic bacteria, gut-associated microorganisms, and engineered microbial systems have expanded the potential industrial and nutraceutical applications of these biocatalysts. Representative recent studies published between 2024 and 2026 are summarised in Table 4.

Collectively, these recent studies demonstrate a clear shift from traditional applications of α-L-rhamnosidases towards highly engineered, application-oriented biocatalytic systems. Current research increasingly focuses on improving thermostability, acid tolerance, solvent compatibility, recombinant productivity, and regioselective flavonoid conversion. Furthermore, advances in immobilisation technologies, synthetic biology, and high-density fermentation strategies are accelerating the industrial implementation of microbial α-L-rhamnosidases for sustainable food processing, targeted nutraceutical production, and precision biotransformation processes.

## 4. Genetic Organization and Regulation

α-L-rhamnosidase genes are frequently associated with carbohydrate transporters, transcriptional regulators, and enzymes involved in L-rhamnose catabolism [5,40]. In *L. plantarum*, rhamnosidase loci are often organised with permease genes and regulatory elements, linking substrate uptake to intracellular metabolism [5,11,15].

The rhaP2B2P1B1 locus of *L. plantarum* NCC245 is a representative example of a gene cluster controlled by induction and carbon catabolite repression [15].

Carbon catabolite repression is a key regulatory mechanism. Glucose represses α-L-rhamnosidase activity and transcription of rhamnosidase-associated genes, whereas L-rhamnose induces expression [15]. Similar regulatory logic has been reported in fungi, where L-rhamnose induction and glucose repression influence the expression of α-L-rhamnosidase genes and uptake systems [40,41]. These regulatory features are important for food fermentations, where carbon source composition may determine whether a strain expresses the desired rhamnosidase activity.

Transport is another major determinant of whole-cell performance. Several enzymes exhibit high catalytic activity in cell extracts but limited conversion in intact cells because bulky flavonoid glycosides are poorly internalized [5,10,34]. Engineering transporters, permeability, or extracellular secretion may therefore be necessary to develop efficient whole-cell biocatalysts for flavonoid transformation. Although transporter systems involved in flavonoid uptake remain poorly characterized in many microorganisms, increasing evidence suggests that membrane transport constitutes a major limiting factor in whole-cell biocatalysis. In addition to passive diffusion, substrate uptake may involve specific permeases, porins, or ATP-dependent transport systems whose expression varies among microbial species. Future engineering strategies may therefore benefit from combining α-L-rhamnosidase overexpression with targeted optimization of transport functions, membrane permeability, and extracellular enzyme display systems. A better understanding of transport-related bottlenecks will be essential for improving flavonoid conversion efficiency in food-grade and industrial microbial platforms.

## 5. Substrate Specificity and Selective Deglycosylation

Substrate specificity is a defining feature of microbial α-L-rhamnosidases. Comparative regioselective behaviours towards α-(1→2)- and α-(1→6)-linked substrates are summarised in Table 5. These enzymes can discriminate between alpha-(1→2), alpha-(1→3), alpha-(1→6), and direct alpha-O-rhamnosyl linkages, and their activity may also depend on the aglycone scaffold [5,6,14].

This explains why activity on p-nitrophenyl-alpha-L-rhamnopyranoside does not necessarily predict activity on natural flavonoids [10,13,42]. Selective deglycosylation is technologically important because many applications require the accumulation of mono-glycosylated intermediates rather than complete hydrolysis to aglycones [6,7,10]. Rutin can be converted to quercetin-3-O-glucoside (isoquercitrin), naringin to prunin, hesperidin to hesperetin-7-O-glucoside, and neohesperidin to hesperetin derivatives [6,7,10]. These products are valuable because they may exhibit improved solubility, intestinal absorption, and biological activity [7,43].

Commercial enzyme preparations often contain both α-L-rhamnosidase and beta-D-glucosidase activities. This can be beneficial for complete debittering but problematic for the selective production of mono-glucosides [7,10,24]. Thermal treatment, immobilisation, or recombinant single-enzyme production can shift product distribution towards desired intermediates by reducing beta-glucosidase side activity [7,24].

## 6. Analytical and Biochemical Characterization Methods

Rhamnosidase activity is commonly screened using chromogenic or fluorogenic substrates such as p-nitrophenyl-α-L-rhamnopyranoside and 4-methylumbelliferyl-α-L-rhamnopyranoside [1,10,12]. These assays are simple and suitable for rapid screening, but they may underestimate or misrepresent activity towards natural flavonoids [10,42]. HPLC-UV, UPLC-MS, LC-MS/MS, GC-FID, TLC, and MALDI-TOF-MS are widely used to quantify substrate depletion, released sugars, and intermediate products [6,7,10]. The advantages and limitations of the main analytical methods used for α-L-rhamnosidase characterisation are summarised in Table 6. This table offers an overview of analytical, chromatographic, and spectrometric methods commonly employed for the biochemical characterisation of α-L-rhamnosidases, including their principal applications, advantages, and methodological limitations. NMR spectroscopy can further confirm glycosidic linkage positions and product structures. Kinetic characterisation generally includes Km, Vmax, kcat, and kcat/Km values, but comparisons across studies are difficult because assay pH, temperature, substrate concentration, enzyme purity, and detection methods differ substantially [1,2]. Standardised panels containing representative alpha-(1→2)- and alpha-(1→6)-linked flavonoids would improve comparability and industrial relevance.

## 7. Food and Beverage Applications

The best-established application of α-L-rhamnosidases is the debittering of citrus juice. Representative food and beverage applications of microbial α-L-rhamnosidases are summarised in Table 7.

**Figure 3:**
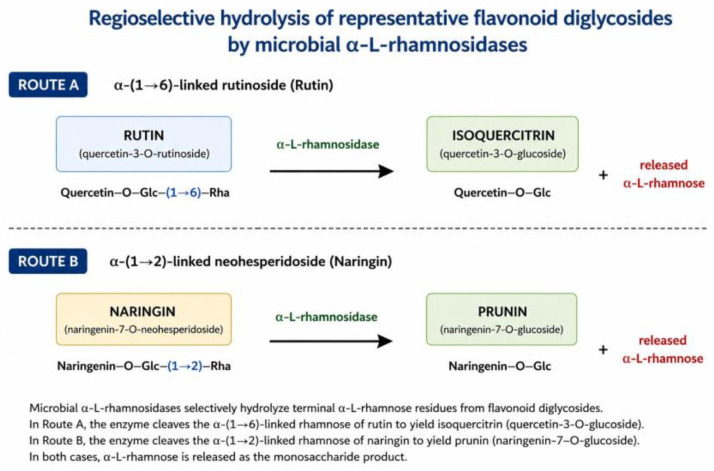
Regioselective hydrolysis of representative flavonoid diglycosides by microbial α-L-rhamnosidases. The enzyme selectively removes terminal α-L-rhamnose residues from α-(1→6)-linked rutinosides and α-(1→2)-linked neohesperidosides, yielding the corresponding mono-glucosylated derivatives isoquercitrin and prunin. Figure created by the author based on information synthesized from the cited literature.

Naringin contributes significantly to the bitterness of grapefruit and bitter orange juice, and naringinase-mediated hydrolysis reduces this sensory defect by converting naringin to prunin and, when beta-glucosidase is present, to naringenin [1,13,17,44]. Enzymatic debittering is attractive because it preserves nutritional and sensory quality better than harsh chemical methods, although acid stability and cost remain important constraints [21]. The regioselective hydrolysis of flavonoid diglycosides represents one of the most important biotechnological applications of microbial α-L-rhamnosidases. These enzymes selectively remove terminal α-L-rhamnose residues from glycosylated flavonoids while preserving the remaining glucose moiety, thereby generating mono-glucosylated derivatives with altered physicochemical and biological properties. Representative examples include the conversion of rutin, containing an α-(1→6)-linked terminal rhamnose residue, into isoquercitrin, and the conversion of naringin, containing an α-(1→2)-linked terminal rhamnose residue, into prunin, as summarized in Figure 3.

In winemaking, α-L-rhamnosidases enhance aroma by hydrolysing glycosylated terpene precursors, releasing volatile compounds such as linalool, geraniol, nerol, and citronellol [1,4,12]. Enzymes compatible with ethanol, low pH, and wine-processing conditions are especially desirable [12]. Functional beverages and plant-based foods are a newer application area. Rhamnosidases can enrich tomato, citrus, tea, and berry products with more bioavailable flavonoid derivatives, and LAB systems may enable in situ transformation during fermentation [5,10,13]. Using citrus solid waste as an inducer and substrate for fungal enzyme production also supports circular bioeconomy approaches [17,26].

## 8. Nutraceutical and Health-Related Applications

α-L-rhamnosidase-mediated deglycosylation can enhance the bioavailability and biological activity of dietary flavonoids [7,16,43]. Representative nutraceutical biotransformations and their associated biological effects mediated by microbial α-L-rhamnosidases are summarised in Table 8.

Rhamnosylated flavonoids such as rutin and hesperidin are poorly absorbed in the small intestine because humans lack endogenous rhamnosidase activity, whereas glucosides or aglycones produced by microbial or enzymatic hydrolysis may be absorbed more efficiently [5,35,43]. Isoquercitrin is a key example. Enzymatic conversion of rutin to quercetin-3-O-glucoside can increase radical-scavenging activity in DPPH assays and markedly enhance antiproliferative effects in cancer cell models compared with rutin [7]. The improved performance of mono-glucosides is partly attributed to intestinal transport mechanisms, including sodium-dependent glucose transporters, although activity remains highly dependent on the assay system, cell type, and metabolic context [7,43]. Gut microbiota strongly influences flavonoid metabolism and health outcomes. Dietary flavonoids can modulate microbial community composition, while microbial enzymes convert glycosides into absorbable metabolites [2,16,36]. This bidirectional interaction supports the concept of microbiome-assisted nutraceutical activation and suggests that rhamnosidase-producing probiotics could help shape individual responses to polyphenol-rich diets [16,35].

Despite promising results, several limitations should be considered when evaluating the nutraceutical potential of α-L-rhamnosidase-mediated flavonoid biotransformation. Most available evidence derives from in vitro studies, cell culture experiments, or animal models, whereas human clinical data remain scarce. Furthermore, considerable interindividual variability exists in flavonoid metabolism due to differences in gut microbiota composition, dietary habits, host genetics, and intestinal physiology. Consequently, improved bioavailability observed under controlled experimental conditions does not necessarily translate into equivalent health benefits in humans. Future studies should prioritize well-designed clinical investigations and microbiome-informed approaches to better understand the real-world impact of α-L-rhamnosidase-derived flavonoid metabolites.

## 9. Probiotic and Food-Grade α-L-Rhamnosidases

Food-grade and probiotic α-L-rhamnosidases are attractive because they combine catalytic activity with safety and compatibility with fermented foods [5,10,15]. Among LAB, *L. plantarum* is the most studied producer and often carries multiple GH78 enzymes with distinct biochemical behaviours [9,11]. These enzymes are particularly relevant for transforming rutin and hesperidin into mono-glucosylated products [5,10,11]. However, whole-cell applications are limited by the transport of bulky flavonoids and by carbon source-dependent regulation of rhamnosidase expression [5,15]. A major bottleneck in using whole-cell LAB biocatalysts is the restricted intracellular uptake of glycosylated flavonoids. Recent genetic approaches address this limitation by fusing α-L-rhamnosidase genes with strong native signal peptides to drive extracellular enzyme secretion, or by utilizing mild, food-grade cell-permeabilizing agents that enhance membrane porosity without compromising cell viability. Future food-grade platforms will likely require coordinated optimisation of enzyme expression, substrate transport, and fermentation conditions.

## 10. Recombinant Production and Enzyme Engineering

Recombinant production is essential for obtaining sufficient quantities of α-L-rhamnosidases with reproducible activity, reduced background glycosidase contamination, and improved industrial applicability [10,29]. *Escherichia coli* remains one of the most widely used hosts for biochemical characterisation and rapid screening of recombinant GH78 enzymes, whereas yeast-based systems such as Pichia pastoris are particularly attractive for secreted enzyme production and large-scale industrial fermentation [17,19,29].

Recent studies have increasingly focused on engineering α-L-rhamnosidases with improved thermostability, acid tolerance, solvent resistance, catalytic efficiency, and regioselective substrate selectivity [38,39]. In particular, thermophilic and acidophilic GH78 enzymes from *Thermotoga* species and hyperthermostable archaeal rhamnosidases have shown strong potential for high-temperature flavonoid biotransformation and citrus juice debittering processes [36,37]. Advances in recombinant engineering strategies have enabled the development of co-expression systems combining α-L-rhamnosidase and β-glucosidase activities for efficient production of bioactive flavonoid derivatives such as quercetin and isoquercitrin [38,39]. Furthermore, high-density fermentation approaches involving molecular chaperones, osmolytes, and optimised cultivation conditions have significantly improved recombinant enzyme yields and industrial scalability [39]. Although whole-cell biocatalysis offers important advantages, including improved enzyme stability and the elimination of costly purification steps, its efficiency is frequently limited by mass-transfer constraints across the cell membrane, which restrict intracellular uptake of bulky flavonoid substrates. To address these limitations, emerging strategies include engineering membrane permeability, co-expression of specific transporters or porins, and the development of surface-display systems that anchor α-L-rhamnosidases to the outer membrane. These approaches can bypass intracellular transport barriers and significantly enhance flavonoid biotransformation efficiency. Structural information obtained from bacterial GH78 enzymes, including those from *Bacillus*, *Streptomyces*, *Klebsiella*, *Bacteroides*, and *Dictyoglomus*, continues to provide valuable templates for rational protein engineering and structure-guided optimization of catalytic properties [4,6,7,19]. Future developments may increasingly integrate synthetic biology, machine learning, and AI-assisted enzyme engineering to facilitate sequence-to-function prediction and support the design of more selective biocatalysts for flavonoid biotransformation.

## 11. Industrial Biocatalysis and Process Engineering

**Figure 4:**
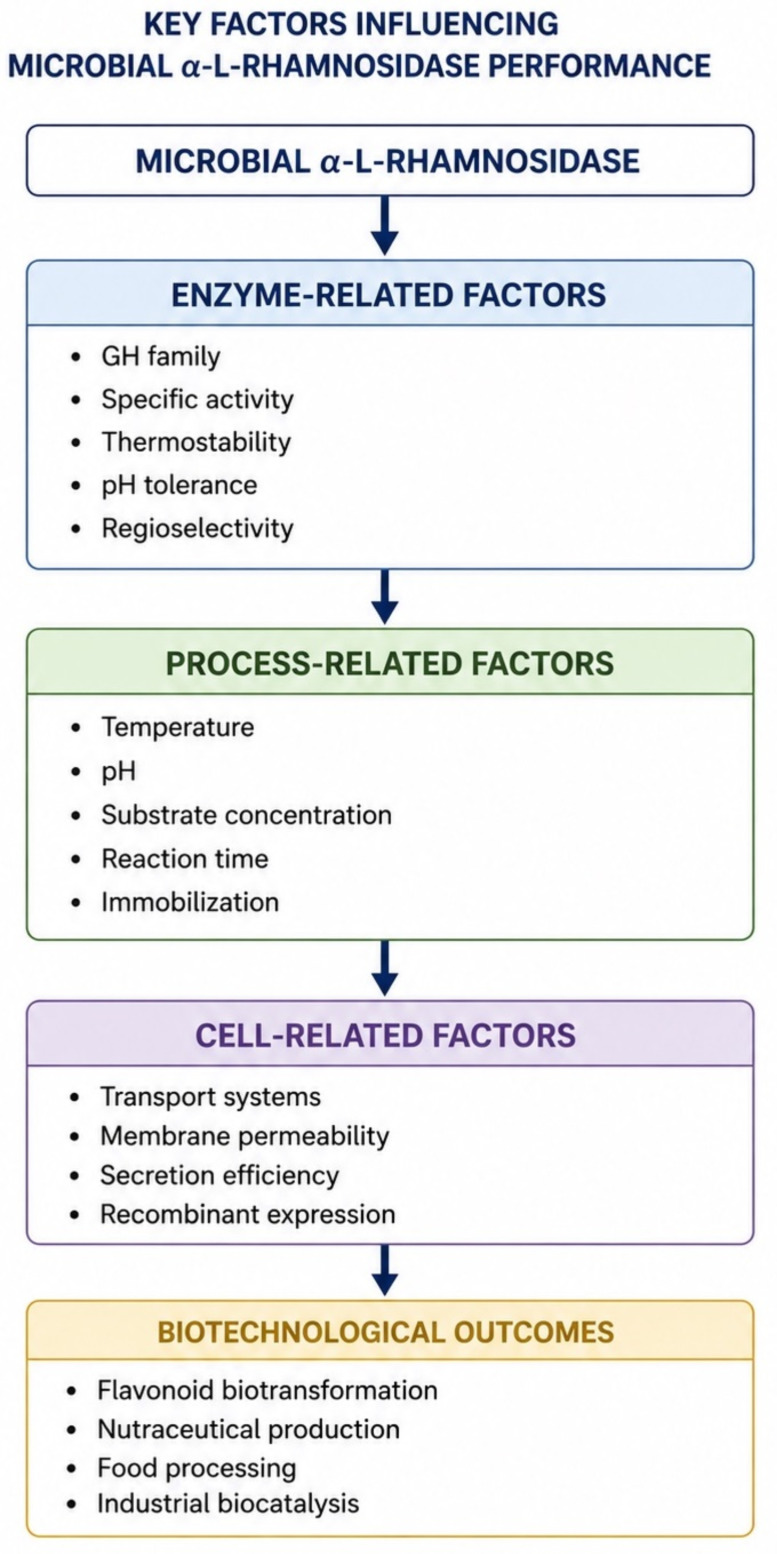
Key factors influencing microbial α-L-rhamnosidase performance in biotechnological applications. Original figure created by the author.

Industrial applications require enzymes that combine selectivity, stability, affordability, and compatibility with real food matrices. Commercial preparations are usually multifunctional glycosidase mixtures, which may improve complete hydrolysis but reduce selectivity for target intermediates [7,10,24]. Recombinant single enzymes and selective inactivation strategies can improve product control. Immobilisation is a major strategy for improving enzyme reuse, stability, and reactor compatibility. Naringinase and rhamnosidase-containing preparations have been immobilised on alginate, chitosan, silica, magnetic supports, polymeric carriers, and activated natural polymers [24,45,46]. Immobilised systems are particularly attractive for continuous debittering, packed-bed reactors, and repeated flavonoid conversion cycles. Sustainability is increasingly important. Enzymatic processes can reduce harsh chemical hydrolysis, operate under mild conditions, and valorise citrus residues as substrates or enzyme inducers [17,26]. Integration with circular bioeconomy strategies could strengthen the industrial relevance of microbial α-L-rhamnosidases. The performance of microbial α-L-rhamnosidases in biotechnological processes is influenced by multiple factors, including intrinsic enzyme properties, process parameters, and cellular characteristics such as transport systems and membrane permeability. These interrelated determinants are summarized in Figure 4.

## 12. Current Challenges and Future Perspectives

Major challenges include limited structural data for fungal and yeast enzymes, insufficient assay standardisation, weak activity in acidic food matrices, poor substrate transport in whole-cell systems, and incomplete understanding of linkage and aglycone selectivity [1,2,4,36]. Current scientific limitations and emerging future perspectives in α-L-rhamnosidase research are summarised in Table 9. This table presents the current scientific and technological limitations affecting the industrial implementation of microbial α-L-rhamnosidases, as well as emerging strategies and future perspectives involving protein engineering, synthetic biology, and precision biocatalysis. Commercial preparations often contain undesired glycosidase side activities, complicating selective production of mono-glycosides [7,10,24]. Future directions include metagenomic discovery of enzymes from gut microbiota and extreme environments, structure-guided engineering, transporter optimisation, immobilised continuous-flow systems, and synthetic biology platforms for integrated flavonoid biotransformation [2,6,16,36]. Computationally assisted enzyme engineering may help improve prediction of substrate specificity and guide future development of selective biocatalysts for nutraceutical production.

## 13. Conclusions

Microbial α-L-rhamnosidases are evolving from traditional debittering enzymes to highly selective biocatalysts for food, nutraceutical, and health applications. Their capacity to selectively remove terminal rhamnose residues from a wide range of natural products enables targeted modification of sensory properties, solubility, bioavailability, and biological activity.

Notably, the regioselective hydrolysis of α-(1→2)- and α-(1→6)-linked flavonoid glycosides has become a key strategy for producing high-value monoglycosylated derivatives such as prunin and isoquercitrin. Recent progress in structural biology, recombinant expression systems, immobilised biocatalysts, probiotic biotechnology, and metagenomic enzyme discovery continues to expand the industrial and biomedical relevance of these enzymes. Simultaneously, increasing evidence linking microbial rhamnosidase activity to gut microbiota-mediated flavonoid metabolism underscores their potential role in personalised nutrition and microbiome-oriented functional foods.

Despite these developments, several challenges persist, including limited structural information for fungal and yeast enzymes, insufficient assay standardization, suboptimal acid and solvent stability, and restricted substrate transport in whole-cell systems. In particular, limited understanding of substrate uptake mechanisms, membrane permeability, and extracellular enzyme accessibility remains a major bottleneck for efficient flavonoid biotransformation.

This review integrates recent advances in GH78/GH106 comparative analysis, flavonoid regioselective biotransformation, microbiota-associated metabolism, transport-related limitations, and emerging engineering strategies within a unified framework focused on microbial α-L-rhamnosidases.

Future progress may benefit from integrated approaches combining protein engineering, synthetic biology, AI-assisted design tools, and transport optimization, although these approaches remain at different stages of development and require further experimental validation. Overall, microbial α-L-rhamnosidases represent valuable tools for sustainable biotransformation, clean-label food processing, targeted nutraceutical production, and next-generation functional food development.

## Figures and Tables

**Table 2:** Comparative Features of GH78 and GH106 α-L-Rhamnosidases.

Feature	GH78	GH106	References
CAZy classification	Glycoside Hydrolase Family 78	Glycoside Hydrolase Family 106	[21]
Catalytic mechanism	Inverting	Inverting	[3,21]
Distribution	Widely distributed in fungi, yeasts, bacteria and thermophiles	Predominantly bacterial	[2,3,21]
Structural information	Multiple crystal structures available	Limited structural characterization	[20,22,23]
Representative enzymes	*Bacillus* sp. RhaB, DtRha, Streptomyces avermitilis Rha	Selected bacterial α-L-rhamnosidases	[6,20,22,23]
Typical substrates	Flavonoid rutinosides, neohesperidosides, terpenyl glycosides	Natural rhamnosides and oligosaccharides	[2,6,14]
Flavonoid biotransformation studies	Extensive	Limited	[6,10,14]
Industrial applications	Citrus debittering, wine aroma release, nutraceutical production	Potential applications, still underexplored	[1,7,12,13]
Biotechnological relevance	High	Moderate	[2,21]
Current research status	Well established	Emerging	[2,3,21]

**Table 3:** Structural and Catalytic Features of Representative Microbial α-L-Rhamnosidases.

Enzyme	MW (kDa)	Oligomeric State	Catalytic Residues	Thermostability	Solvent Tolerance	References
*P. angusta* Rha	90	Monomer	Asp/Glu	Moderate	Low	[18]
DtRha	~100	Monomer	E479/E782	Very high	High	[6]
RhaB1	~80	Homodimer	Asp/Glu	Moderate	Moderate	[20,22]
RhaB2	~85	Homodimer	Asp/Glu	High	Moderate	[23]

**Table 4:** Recent Engineering Strategies and Technological Advances in Microbial α-L-Rhamnosidases (2024–2026).

Study Focus	Technological Innovation	Main Outcome	Industrial Relevance	Reference
Thermophilic GH78 from *Thermotoga* sp.	Acidophilic thermoenzyme	Efficient hydrolysis of flavonoid diglycosides	High-temperature food processing	[36]
Archaeal GH78 rhamnosidase	Hyperthermostable enzyme	Efficient juice debittering	Industrial citrus processing	[37]
*Aspergillus niger* co-expression system	Dual α-L-rhamnosidase/β-glucosidase platform	Improved quercetin production	Nutraceutical manufacturing	[38]
*Escherichia coli* recombinant production	Chaperone-assisted high-density fermentation	Enhanced enzyme yield	Industrial enzyme production	[39]
Human gut bacterial GH78 enzymes	Regioselective flavonoid hydrolysis	Selective conversion of rutin and naringin	Precision nutraceuticals	[14]
Immobilized fungal naringinase	Magnetic polysaccharide carrier immobilization	Improved operational stability	Continuous biocatalysis	[24]

**Table 5:** Regioselective Hydrolysis and Substrate Specificity of Microbial α-L-Rhamnosidases.

Enzyme	α(1→2) Activity	α(1→6) Activity	Preferred Substrate	Main Product	Key References
DtRha	High	Low	Naringin	Prunin	[6]
RhaB1	Low	High	Rutin	Isoquercitrin	[5]
RhaB2	Low	High	Hesperidin	Hesperetin glucoside	[7,10]
Commercial naringinase	Moderate	Moderate	Naringin	Naringenin	[7,13]

**Table 6:** Analytical and Biochemical Methods for α-L-Rhamnosidase Characterization.

Method	Purpose	Advantages	Limitations	References
pNPR assay	Rapid screening	Simple and inexpensive	Poor natural substrate prediction	[1,10]
HPLC	Flavonoid quantification	High accuracy	Longer analysis time	[7]
UPLC-MS	Product identification	High sensitivity	Expensive instrumentation	[6]
MALDI-TOF-MS	Mass analysis	Rapid structural analysis	Specialized expertise	[10]
HPAEC-PAD	Sugar quantification	High selectivity	Complex setup	[2]

**Table 7:** Food Processing and Beverage Applications of Microbial α-L-Rhamnosidases.

Application	Substrate	Product	Industrial Benefit	References
*Citrus* debittering	Naringin	Prunin	Reduced bitterness	[1,13,17]
Wine aroma enhancement	Terpene glycosides	Volatile terpenes	Improved aroma	[4,12]
Functional beverages	Rutin	Isoquercitrin	Enhanced bioavailability	[10,43]
Tomato biotransformation	Rutin	Quercetin glucosides	Nutraceutical enrichment	[7]

**Table 8:** Nutraceutical and Health-Related Applications of Microbial α-L-Rhamnosidases.

Parent Compound	Enzymatic Product	Improved Property	Biological Effect	References
Rutin	Isoquercitrin	Bioavailability	Antioxidant	[7,43]
Naringin	Prunin	Reduced bitterness	Anti-inflammatory	[13,16]
Hesperidin	Hesperetin glucoside	Absorption	Cardioprotective	[10]
Ginsenosides	Minor ginsenosides	Bioactivity	Anticancer potential	[2]

**Table 9:** Current Challenges and Future Perspectives in α-L-Rhamnosidase Research.

Current Limitation	Impact	Possible Solution	Future Perspective	References
Poor acid stability	Reduced food-processing efficiency	Protein engineering	Acid-stable enzymes	[24,36]
Limited structural data	Restricted rational design	Cryo-EM and crystallography	Structure-guided engineering	[2,22,23]
Low substrate transport	Reduced whole-cell catalysis	Transport engineering	Engineered probiotics	[5,34]
Mixed enzyme specificity	Undesired by-products	Selective biocatalysts	Precision nutraceuticals	[7,10,24]

## Data Availability

No new data were created or analysed in this study. Data sharing is not applicable to this article.
